# An Evidence-Based Education Program for Adults about Child Sexual Abuse (“*Prevent It!*”) That Significantly Improves Attitudes, Knowledge, and Behavior

**DOI:** 10.3389/fpsyg.2016.01177

**Published:** 2016-08-19

**Authors:** Erin K. Martin, Peter H. Silverstone

**Affiliations:** Department of Psychiatry, University of AlbertaEdmonton, AB, Canada

**Keywords:** child, sexual, abuse, adult, education, prevention, program, evaluation

## Abstract

Here we describe the development of an evidence-based education program for adults about childhood sexual abuse (CSA), called *Prevent It!* Uniquely, the primary goal of this program was to change the behavior of participants, as well as to increase knowledge about CSA and positive attitudes toward it. A comprehensive review shows no previous similar approach. The program includes a detailed manual to allow standardized administration by trained facilitators, as well as multiple video segments from CSA survivors and professionals. A total of 23 program workshops were run, with 366 adults participating. Of these, 312 (85%) agreed to take part in the study. All completed baseline ratings prior to the program and 195 (63% of study sample) completed follow-up assessments at 3-months. There were no significant differences between the demographic make-up of the baseline group and the follow-up group. Assessments included demographic data, knowledge, attitudes, and several measures of behavior (our primary outcome variable). Behavioral questions asked individuals to select behaviors used in the previous 3-months from a list of options. Questions also included asking “how many times in the previous 3-months” have you “talked about healthy sexual development or Child sexual abuse (CSA) with a child you know”; “suspected a child was sexually abused”; “taken steps to protect a child”; or “reported suspected sexual abuse to police or child welfare”? The majority of attendees were women, with the commonest age group being between 30 and 39 years old. Approximately 33% had experienced CSA themselves. At 3-month follow-up there were highly statistically significant improvements in several aspects of behavior and knowledge, and attitudes regarding CSA. For example, the number of subjects actively looking for evidence of CSA increased from 46% at baseline to 81% at follow-up, while the number of subjects who actively took steps to protect children increased from 25% at baseline to 48% at follow-up. For general public adults, this is the first program designed using the current evidence base for effective training in CSA examining longer-term outcomes and the first to focus on actual behavioral outcomes. The results suggest it is highly effective and support its widespread use.

## Introduction

Child sexual abuse (CSA) is common (Finkelhor, 1994; Pereda et al., 2009), with as many as 1 in 6 girls and 1 in 12 boys currently experiencing sexual abuse involving bodily contact (Martin and Silverstone, 2013). It is uncertain if there are changes in the rates of CSA in the United States (Finkelhor, 2009) or Canada (Collin-Vézina et al., 2010), particularly given the increase in use of internet pornography (Diamond et al., 2011; Livingstone and Smith, 2014). Adults who experienced CSA have higher rates of psychopathology (Chen et al., 2010) and are at higher risk of a range of medical, psychological, behavioral, and sexual disorders (Maniglio, 2009) than those who have not been sexually abused. It is possible that these impacts are mediated through changes in brain structures and functions (Anda et al., 2006; Kaffman, 2009; Belsky and de Haan, 2011), as well as neurobiological stress systems, with “child maltreatment (being) a preventable contributor to child psychopathology, cognitive impairment, and developmental difficulties” (Watts-English et al., 2006).

Because of the frequent long-term impacts of CSA, preventing it is of great importance. One method is to include a range of people in prevention efforts including parents, professionals, and the general public (Wurtele, 2009). Toward this end, it is likely that adults who interact with children may be in the best position to reduce children's vulnerability to CSA, as well as to reduce their vulnerability to negative impacts associated with CSA. This is of value as positive adult support may improve outcomes, helping children experience fewer negative impacts than others (Yancey et al., 2011). It should also be recognized that previous research into the possible effectiveness of adult training programs has provided insight into knowledge gain, attitude change, and behavioral *intentions* (Rheingold et al., 2007; Self-Brown et al., 2008), but not on the key issue of actual behavior *changes* in regards to CSA preventative behaviors.

There is great variety in the types of prevention programs that exists, their target groups, the methods, and their specific contexts (Zollner et al., 2014). Despite this variety, there are few CSA prevention programs targeting general public adults that have been developed using a research informed approach, and none to our knowledge that reflect recent research findings. All such programs do need to be based on current theoretical understandings of healthy child development (Zeuthen and Hagelskjær, 2013). Furthermore, evidence-based approaches that include policy, legislation, and services are also currently lacking (Collin-Vézina et al., 2010). For this reason, we believe that development of a novel program is required, and here we report on such a program, titled: *Prevent It!* Taking Action to Stop Child Sexual Abuse (*Prevent It!*). In the current publication we describe the current research background, the theoretical background, methodology of development of the program, and the first study into it's effectiveness. In particular, we examined the extent to which a classroom-based CSA prevention program for adults can change participant's longer-term behaviors, as well as attitudes and knowledge.

## Materials and methods

### Ethical and personal impact consideration

This study was approved by the Ethics Board of the University of Alberta. All participants completed informed consent prior to any involvement in the program. All individuals were given a unique study number, and this was the only identifier used to collect all study-related information at a secure on-line site. To ensure confidentiality for participants in a group setting we only used first names, and also included a section in the introduction of the program where the facilitator asks participants to respect and maintain each other's confidentiality.

Additionally, there is potential for the program to be emotionally upsetting, and have a personal impact particularly for those individuals who have themselves experienced CSA. We therefore took several steps to address this concern. Firstly, all participants were provided with a 24-h crisis line resource to access, should they want to talk to someone outside their own support system or get more information about professional services. Secondly, the facilitators discussed this issue at the start of the program, letting each participant know that the program could be upsetting to them, or could bring up thoughts and feelings that are difficult to manage. Thirdly, participants were encouraged to use their own support system, if needed, and were reminded of this at the start and end of the program.

### Program development and literature review

We reviewed literature related to CSA topics in order to determine program content using the databases PubMed, PsycINFO, and ERIC. In order to obtain an overview of the topic we searched the databases using the following search terms: “sexual abuse,” “rates,” “occurrence,” “epidemiology,” “impact,” “signs,” “indicators,” “victim,” “survivor,” “offenders,” “perpetrators,” “disclosure,” “education,” “prevention,” “reporting,” “parents,” “children,” “caregivers,” and “teachers.”

### Current situation

School-based, child targeted prevention programs have been widely used in CSA education (Zwi et al., 2007; Wurtele, 2009; Walsch et al., 2015). Accessing children directly through schools allows educators to reach children from a wide range of socio-economic and ethnic backgrounds (Wurtele, 2009). Targeting children at school rather than relying on parents to provide the information helps ensure children who are being abused in their homes still have access to CSA education. The extent to which schools are currently using this type of approach is yet to be assessed in a systematic way (Wurtele, 2009). Literature reviews (MacMillan et al., 2009; Fryda and Hulme, 2015) and meta-analysis (Davis and Gidycz, 2000; Zwi et al., 2007; Walsch et al., 2015) indicate child-targeted programs can increase children's knowledge, self-protective behaviors, and reporting behaviors (MacMillan et al., 2009). While child-targeted CSA education plays an important role in CSA prevention, adults may also be a useful target group (Wurtele, 2009; Zeuthen and Hagelskjær, 2013).

Of the adult-targeted education, research on caregivers is most common (Berrick, 1988; Hébert et al., 2002; Rheingold et al., 2007; Self-Brown et al., 2008; Kenny, 2009). Caregivers may be in positions where they can rapidly identify CSA situations and help at-risk or affected children (Hébert et al., 2002). It should be noted that children's disclosure of abuse is often a process rather than a one-time event (Alaggia, 2004; Jenson et al., 2005), and their caregivers can be in ideal positions to respond to these disclosures. Nonetheless, a caregiver may also be involved in perpetrating abuse (Public Health Agency of Canada, 2010), making it necessary for adults outside the home to also receive CSA education. In part for this reason, it has been suggested (Wurtele, 2009) that non-caregiver adults should also have CSA education, although these adults remain the least studied group. Teachers have received slightly more attention than other non-caregiver adults (Walsch et al., 2012), and the potential effectiveness of media campaigns on general adult populations has also been examined (Self-Brown et al., 2008). The focus of many adult-targeted education programs has been on changing knowledge and attitudes about CSA (Bowman et al., 2010; Walsch et al., 2012), as well as behavioral *intentions* with regard to responding to suspected CSA (Hébert et al., 2002; Self-Brown et al., 2008). To our knowledge, there are no previous studies that have examined actual changes in behavior after training programs.

Adult-targeted CSA education programs use a variety of formats to teach adults about CSA. One randomized controlled study measured attitudes, knowledge, and behavioral intentions after being exposed to media-based CSA materials founding a significant increase in knowledge scores after exposure but no significant change in attitude (Rheingold et al., 2007). Another study used survey and focus group methods to evaluate the effectives of public service announcements on knowledge and likelihood of behavioral change, but while they found a very short-term increase in knowledge, participants agreed this approach was not enough to change behavior (Self-Brown et al., 2008). The results from these studies suggest that a brief exposure to CSA educational materials may increase knowledge in the short-term, but not be sufficient to change behavior. One online version of a CSA education program shows that professionals (i.e., teachers, psychologist, and social workers) who interact with children and youth regularly had significant gains in CSA related empathy in the 6 months after taking the online program (Man-Ging et al., 2015). Similarly, other classroom-based studies show an increase in short-term knowledge as well as increase appropriate behavioral intentions (Hébert et al., 2002; Kenny, 2009; Bowman et al., 2010; Rheingold et al., 2012). However, only one of these studies included follow-up assessment beyond 1 month (Rheingold et al., 2015), and thus the longer-term effectiveness of previous adult-targeted classroom-based education is promising but far from certain. It is also unclear if repeated information sessions, rather than a single exposure, alters the effectiveness of education (Davis and Gidycz, 2000; Rheingold et al., 2007). According to the results of a multi-site, randomized control trail study, adult-targeted CSA education programs designed for childcare professionals can increase knowledge and self-reported use of prevention behaviors in both classroom and online versions (Rheingold et al., 2015). There are no studies that show an increase in knowledge over more than a 1-month period, and none that have examined actual behavioral changes in general public adults. Given increasing literature and knowledge about the most effective types of adult education (Petty and Thomas, 2014; Taylor and Laros, 2014), we developed a novel program education program targeting general population adults (*Prevent It!*) to address this issue. We also evaluated the program, with our primary outcome measure focusing on behavior changes over a 3-month period.

### Consultation and theoretical grounding

Taking into consideration the current situation, we consulted with topic experts in order to assess current needs (and potential gaps) in CSA education with a wide group of individuals. These included members of the Police Services involved with all aspects of CSA, psychologists working in the field of CSA, charities involved in training, and with sexual assault centers. Because our main outcome goal for the program was behavior change, it was important to structure the program utilizing methodology previously demonstrated to maximize likelihood of subsequent behavioral changes. Although some studies have shown changes in behavior *intentions* after taking an adult-targeted CSA education program (Hébert et al., 2002; Rheingold et al., 2007; Self-Brown et al., 2008; Paranal et al., 2012) and one study measured self-report behavior change in childcare professionals (Rheingold et al., 2015) we are unaware of any studies measuring actual behavior changes in general public adults. The lack of research means that little is known about the key components required to change participant's behavior in regards to sexual prevention. For these reasons, we used previously well-established models, namely the Transtheoretical Model of change (TTM) (Diclemente et al., 1991; Diclemente and Prochaska, 1998) and the Experiential Learning Cycle (Kolb, 1975) to develop the program.

The TTM approach to understanding behavior change combines all components into a single cohesive theory (Diclemente and Prochaska, 1998). Although initially developed to understand behavior in regards to addictions, the TTM is useful in helping understand how adults change their behavior. According to the TTM model, adults cycle through five stages when changing their behavior. In (1) *pre-contemplation*, adults are not yet considering the behavior change in question, while in (2) *contemplation*, the person considers change but has not yet decided to make the change. In (3) *preparation*, the person prepares for, and plans for, making the change and in (4) *action*, the individual starts making the change. Finally, in (5) *maintenance*, the change has is made and is continued over a longer period of time. The program was therefore designed to facilitate the movement of participants through each subsequent stage, and to assist them to reach the action (stage 4) and maintenance (stage 5) phases of change.

The activities in the program were structured utilizing methodology described in the Experiential Learning Cycle (ELC) (Kolb, 1975). The ELC can be considered as a tool used to create movement through the TTM. This cycle has 4 components: (1) concrete experience, (2) reflective observation, (3) abstract conceptualization, and (4) action/active experimentation. First, the facilitator introduced a structured experience, such as playing a video clip segment or directing participants to an activity in the program. Experiences engage participants in ways that relate to the program's desired outcomes. They also provide participants with a common base for discussion. Second, the facilitator guides participants to reflect on the previous concrete experience encouraging then to personalize the experience by exploring their feelings about, reactions to, and observations of the activity. Third, the facilitator poses “*so what*” and “*now what*” questions to help participants look for personal lessons and future applications. In this step participants make real-world meaning of the activity by utilizing more general principals. Finally, the facilitator helps participants explore ways in which they can implement their learning in their own lives and organizations. They are encouraged to think creatively about the ways in which these leanings can be applied in their unique situations as well as consider possible barriers to implementing changes. Utilizing the well-validated TTM and the ELC ensured the strong theoretical foundation for the program.

#### Content and program guides

Program content was determined after the detailed consultation process, combined with regular communication and contact with the range of stakeholders and also with experienced trainers. A detailed “program guide” was developed, including the rationale for each section, presented in an easy to understand format written for a general audience. It provides all necessary background information for the program content and approach. The program guide is an 80-page document and was made available to program staff.

A briefer “information book” was also developed for all program participants, derived directly from the program guide, to be used by all participants during the program, and subsequently by them as reference material. During the program, facilitators directed participants to the relevant sections of the information books to engage in exercises throughout the program, and used this to guide participants through the exercises.

The program includes a significant video component, with the group facilitator showing video segments throughout the program. The facilitator introduces each section before showing it, and after viewing each segment, there was a guided exercise aimed to increase personal and critical reflection on the material. Video sections include overall narration, interviews with experts in the area, survivors of sexual abuse, and professionals who work in related fields.

The survivor interviews provide personal accounts of sexual abuse as well some of the impact of CSA on them in both the shorter-term and longer-term. These interviews helped to highlight the personal toll of CSA and remind participants that CSA significantly impacts of range of different people in multiple ways. In order to reduce the potential for harm to people that might regret their decision to tell their story in the educational video format, only CSA survivors who were already publicly sharing their stories were approached to be involved. All survivors were told in advance about the types of questions they would be asked in the interview, and were told that they were in control of the question answering process. They did not need to answer all the questions if they did not want to, and they were reminded that they could change their mind and decide not to participate at any point in time during the video production phase or subsequently.

Professionals working in fields related to sexual abuse were also interviewed. Effort was taken to have a wide range of professional voices represented. Interviews featured in the film, in addition to CSA victims, include a psychologist and Traumatologist, an experienced police detective involved in many cases of CSA, a defense attorney, and a psychiatrist.

#### Facilitator guide

The facilitator guide explained what was expected from the facilitator at each point in the program workshop, with information grouped around several themes. Table 1 outlines program themes and topics. The program workshops were designed to be given by community volunteers, rather than specialists, to allow it to be generalizable. For this reason, the Facilitator Guide was written in non-specialist language. Additionally, this model allows for volunteers from a range of communities to be trained and to return to their own communities (and surrounding areas) to provide the program. We believe this model can increase access to the program for individuals in many areas, is scalable, and also practical for use in remote and rural areas. In order to allow for volunteers to facilitate the program consistently we created a highly detailed guide for them to follow. The guide outlines what to say to introduce each video segment and the subsequent exercises. It also provides questions to prompt participant reflection, and identifies key points that are to be emphasized or summarized at the end of each exercise. In the present study we utilized two individuals who received funding for running the program workshops, and who had significant experience doing this previously. To ensure fidelity across sessions, facilitators were instructed to follow the script and guide closely during each program workshop.

**Table 1:** **Content of the adult-targeted child sexual abuse education program**.

**Section**	**Topics**
General Information	• Definitions • Rates • Offenders • Internet
Talking with Children	• Being a good listener • HSD^a^ • Talking with children about HSD • Talking with children about CSA^b^
Observing children and adults	• Possible signs of distress in children • Possible signs of CSA • Grooming • Concerning signs in other adults
Preparing for Action	• Disclosures of CSA • Reporting CSA • Suspicions of CSA • Individual prevention strategies • Organizational best practices • Individualized goal setting

*General sections and the topics included within the sections for the Prevent It! child sexual abuse education program*.

a *HSD, healthy sexual development*.

b *CSA, child sexual abuse*.

### Program evaluation

The target group was adults who interact with children, including caregivers, teachers, coaches for sports and recreation activities, youth group leaders, and religious leaders. This study evaluated the effectiveness of the *Prevent It!* program at changing adult participant's behaviors 3-months after the training session (the primary outcome goal), as well as improving attitudes toward CSA and knowledge about this. A priori hypotheses were that participants who took the workshop would: (1) decrease adherence to problematic myths about CSA (referred to as negative attitudes), (2) increase accurate knowledge about CSA, and (3) increase their use of individual and organizational prevention behaviors to reduce risk of CSA and identify this early. One explicit long-term goal, not studied in the current program, was that this program will help with early identification, and appropriate interventions, of CSA when it occurs. We hope to explore this issue in subsequent research.

#### Sampling strategy and setting

We wanted to measure the effectiveness of the program based on the population that it will be serving. To do this, we used a convenience sample of adults who registered to take educational programs with a charity that provides this (Little Warriors).

#### Recruitment

Study participants were recruited from people that enrolled to take the program. While registering for the program, each person was informed in general terms about the study, and asked if he/she could be contacted with more information. Individuals who agreed to find out more were provided with information and the informed consent form. If they agreed to take part they were then given a unique user number, and information about how to use this to access the secure on-line site where information was collected at baseline. The only required inclusion criteria, other than agreeing to take part in the training session and study, was that they were aged 18 or older. There were no additional exclusion criteria for this study.

#### Program implementation

There were a total of 23 program workshops, involving 366 participants, carried out during the final 3-months of 2014 in 14 communities in Western Canada. The individual who facilitated the vast majority of groups had no previous expertise in this, and no background in medicine or allied professions, to allow some approximation toward the anticipated outcomes when given by other similar individuals. Groups who participated in program workshops included those offered to the general public (14), child/youth serving organizations (5), community groups (2), a mother's group (1), and a group of individuals attending a conference regarding youth issues (1).

#### Measurement of effectiveness and research design

Participants were given pre-test (baseline) measurements to assess attitudes, knowledge, and behaviors related to CSA during the 7-days prior to the program workshop. Three months after their participation in the program workshop they were asked to complete follow-up questionnaires. Each participant's post-test results were compared to baseline results to assess the amount of change that occurred. The main hypothesis being tested was that, at 3-months post-training, participation in the *Prevent It!* program would increase behaviors to reduce children's vulnerability to CSA.

#### Procedures

During the 7-days prior the program workshops, participants completed baseline questionnaires via the secure online site. Participants then took part in the 3-h program workshop. Three months after completion of the program, study participants were contacted and asked to complete the follow-up questionnaire online. This process involved contact (via phone or email—at the choice of the participant). A maximum of three attempts were made for each individual who did not complete the follow-up questionnaire.

#### Sample and demographics

Participants were asked to self-report their gender, age, highest education level completed, level of previous CSA training received, and if they had experienced any type CSA themselves during their childhood. Before being asked about their own experience of sexual abuse, each participant was reminded of the confidentiality of their responses and of the reason for collecting the information. Participants were able to skip demographic details if they chose.

The sample consisted of 312 adults who took the program and completed the informed consent and pre-test questionnaire. Of these, 209 individuals (67%) also completed the 3-month follow-up questionnaire. However, of these a total of 12 questionnaires were not usable due to incomplete identifying information, while one individual with the same identifying information completed the questionnaire twice and both versions were therefore excluded. Thus the final study sample consisted of 195 individuals (63%) for whom there was study data, although some individuals did not complete every section as specified below. Demographic details for the 3 month follow-up group are shown in Table 2.

**Table 2:** **Demographics of Study Participants (*n* = 195)**.

**Demographic Information**	**N (%)**
**GENDER**	
Male	21 (11%)
Female	174 (89%)
**AGE**	
18–29	57 (29%)
30–39	64 (33%)
40–49	41 (21%)
50–59	21 (11%)
60 and older	12 (6%)
**LEVEL OF EDUCATION COMPLETED**	
High school	25 (13%)
Post-secondary	139 (71%)
Graduate studies	31 (16%)
**PREVIOUS CSA^a^ TRAINING**	
None	105 (54%)
Some	80 (41%)
Extensive	10 (5%)
**PREVIOUS PERSONAL HISTORY OF CSA**	
Yes	62 (32%)
No	115 (59%)
Unsure	14 (7%)
Did not answer	4 (2%)

*Demographic make-up of the post-test sample. There was no significant difference between the sample at baseline and the sample at 3-month-follow up*.

a *CSA, child sexual abuse*.

It is important to note that we have baseline demographic data on 312 individuals, but detailed follow-up data only 195 individuals. However, a Wilcoxon signed-rank test showed no statistically significant demographic differences between these groups.

#### Knowledge, attitudes, and behavioral change

Previous studies demonstrate increased accurate knowledge and positive attitudes after exposure to a CSA education program (Hébert et al., 2002; Rheingold et al., 2007; Self-Brown et al., 2008; and Bowman et al., 2010). Consequently, we measured these constructs briefly. Knowledge was measured using three Likert scale items. To measure attitudes we utilized three items from the *Child Abuse Myth Scale* (Collings, 1997). Participants were asked to indicate the extent to which they agreed with the statements (Table 3) using Likert scale answers.

**Table 3:** **Measurement of knowledge and attitude**.

**Measure**	**Statement^a^**
Knowledge	Children are most commonly sexually abused by people who are known to the child and the child's family.
	When a child tells an adult he/she was sexually abused, it is important for the adult to determine whether or not the abuse happened.
	If I suspect that a child is being sexually abused, I have a legal obligation to report this abuse to child social services or police.
Attitude	Children who act in a seductive manner are not to blame if an adult responds to them in a sexual way.
	Sexual contact between an adult and a child that does not involve actual or attempted sexual intercourse is unlikely to have serious psychological consequences for the child.
	Children who do not report ongoing sexual abuse must want the sexual contact to continue.

a *Measured using a 5-point Likert scale: Strongly disagree (coded 4), disagree (coded 3), not sure (coded 2), agree (coded 1), strongly agree (coded 0)*.

To measure behavioral change we created questions based upon current best knowledge about how to decrease children's vulnerability to CSA (Martin and Silverstone, 2013). We measured behaviors related to four major categories: talking about CSA and healthy sexual development (HSD); suspecting and reporting CSA; individual action strategies; and organizational action strategies. We assessed participant's use of behavioral strategies by asking them to select the number of times they had used the strategy in the previous 3-months as well as to select all of the specific items they used from provided checklists. Table 4 details the format, measurement, and coding of each question.

**Table 4:** **Measurement and coding of behavior**.

**Behavioral Measures**	**Measurement**	**Codes**
**TALKING ABOUT CSA AND HEALTHY SEXUAL DEVELOPMENT**		
In the past 3-months, how many times have you talked about healthy sexual development or child sexual abuse with children that you know?	0 times	0
	1–2 times	1
	3–4 times	2
	5 or more times	3
In the past 3-months, which of the following have you talked about with a child you know? Select all that apply. • Boundaries • Identifying a range of emotions • Internet safety • Proper names for genitals • Using the word “surprise” for things like birthday presents rather than “secret” • Definition of sexual abuse • Grooming techniques adults might use • Children are never to blame if they are sexually abused • What to do if you are sexually abused • How to tell someone if you are sexually abused • Saying “no” is allowed	Total score between 0 and 11	0 = No 1 = Yes
**SUSPECTING AND REPORTING CSA**		
In the past 3-months, how many times have you suspected a child you know might have been sexually abused?	0 times	0
	1–2 times	1
	3–4 times	2
	5 or more times	3
In the past 3-months, how many times have you reported a child who you suspected was sexually abused to child social services or police?	0 times	0
	1–2 times	1
	3–4 times	2
	5 or more times	3
**INDIVIDUAL ACTION STRATEGIES**		
In the past 3-months, what things have you done individually? Select all that apply. • Watched for signs of abuse in children • Taken steps to protect children from sexual abuse • Been a responsible role model for other adults in your interaction with children	Total score between 0 and 3	0 = No 1 = Yes
**ORGANIZATIONAL ACTION STRATEGIES**		
In the past 3-months, what things has your organization done with adults who interact with or want to interact with children? Select all that apply. • Does not apply to me^a^ • Criminal record checks • Child welfare checks • Screening interviews • Reference checks	Total score between 0 and 8	0 = No 1 = Yes
•Provide written policy outlining appropriate conduct with children • Monitoring one-on-one time between adults and children • Provide written policy for handling suspicions of abuse • Provide written policy for handling disclosure of abuse • Provide written policy for identifying and handling inappropriate comments and behaviors by adults		

a *Participants who selected does not apply to me were excluded from this analysis*.

### Statistical analysis

In order to assess change from pre-test to 3-month follow-up we computed scores for several of the measures. To assess attitude and knowledge change, each Likert scale response was scored using the ideal responses with higher scores representing more ideal responses. Each participant was given an attitude score and a knowledge score at baseline and at pre-test with higher scores representing more ideal responses. These scores were used to determine attitude and knowledge change.

We assessed behavior change in several ways. First, the measures for the number of times talking about CSA and HSD, the number of times suspecting CSA, and the number of times reporting CSA were compared directly between baseline measurement and post-test measurement. Total scores for CSA and HSD related topics, individual action strategies, and organizational action strategies were computed by adding together each strategy selected. We then computed an overall behavior score for each participant at baseline and post-test by summing the scores for CSA/HSD related topics talked about, individual action strategies, and organizational action strategies used to produce a single total score for each participant. Note that for participants who selected *not applicable* for organizational action strategies were excluded from the organizational strategies analysis.

We used the Wilcoxon signed rank test to determine within-subject statistically significant change from baseline measurements to post-test measurements, given that the Wilcoxon test is appropriate for use with non-parametric data and related groups. Note that we did not apply a Bonferroni correction for multiple comparisons since, while a Bonferroni correction can be useful for reducing the risk of Type I errors in multiple comparisons, it also creates a loss of power to detect changes that are present making its usefulness disputed in studies such as the current one (Keppel, 1991; Perneger, 1998; Divine et al., 2013). Effect sizes were calculated using the standardized *z*-score from the results of the Wilcoxon signed Rank test divided by the square root of “n” (Field, 2009). Effect sizes were understood using Cohen's (1992) guidelines: *r* = 0.010 (small effect), *r* = 0.30 (medium effect), *r* = 0.50 (large effect). Statistical significance was defined as *p* < 0.05. Spearman's correlation was used to determine associations between demographic variables and dependent variables. Missing values due to lack of response to individual questions were excluded from analysis.

## Results

### Knowledge and attitude

**Figure 1:**
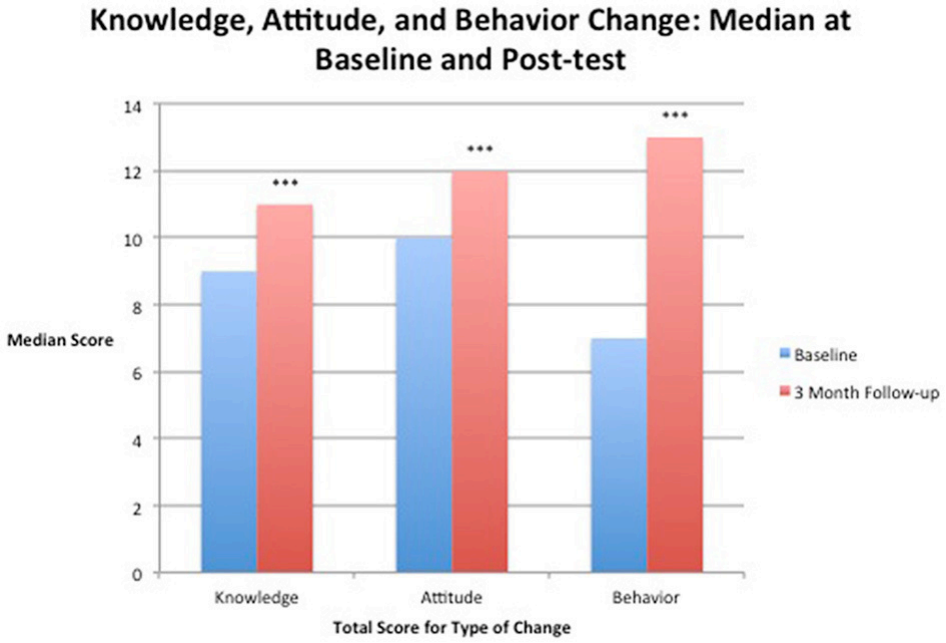
**Median score for attitude, knowledge, and behavior before taking the workshop (baseline) and at 3-months after taking the workshop (3-month follow-up)**. ^***^Results significant at *p* < 0.001.

Measurements of knowledge and attitude had statistically significantly increases from pre-test to 3-month follow-up (Figure 1). A total of 174 study participants completed both baseline and 3-month follow-up questionnaires in the knowledge section of the questionnaire. A Wilcoxon signed-rank test determined a highly statistically significant median increase in knowledge score from pre-test (9) to 3-month follow-up (11), *Z* = 7.463, *p* < 0.001 (Table 5). Of the 174 participants, 108 had higher scores. 44 had no change, and 22 had a decrease. A total of 189 study participants completed both baseline and 3-month follow-up questionnaires in the attitude section of the questionnaire. A Wilcoxon signed-rank test also showed a highly statistically significant median increase in attitude score from pre-test (10) to 3-month follow-up (12), *Z* = 4.724, *p* < 0.001 (Table 5). Of the 189 participants, 78 had increased scores, 86 had no change, and 25 had a decrease in scores.

**Table 5:** **Results for attitude and knowledge change**.

	**Median**		**Range**		***Z*-score**	***p*-value**	**Effect size^a^**
	**Baseline**	**3 Month Follow-up**	**Baseline**	**3 Month Follow-up**			
Total Knowledge Score	9	11	12	7	7.463	<0.0005^***^	0.57
Total Attitude Score	10	12	12	8	4.724	<0.0005^***^	0.34

a *Effect sizes based on Cohen's (1992), r = 0.01 (small effect), r = 0.30 (medium effect), r = 0.50 (large effect)*.

*** *Results significant at p < 0.001*.

### Behavioral measures

All 195 study participants completed at least one section of the behavioral questionnaire at both baseline and follow-up, while a total of 117 study participants completed both baseline and 3-month follow-up questionnaires on all sections of the behavioral questionnaire (including organizational—which didn't apply to many individuals). Results of a Wilcoxon signed-rank test found a highly statistically significant change between the median at baseline and 3-month follow-up, *Z* = 5.322, *p* < 0.001 (Figure 1). From the total number of 113 participants 77 scores increased, 7 scores did not change, and 29 scores decreased.

#### Suspecting child sexual abuse

A total of 186 study participants completed both baseline and 3-month follow-up questionnaires in this section of the behavioral questionnaire. Results of a Wilcoxon signed-rank test found no statistically significant changes between the median at baseline and 3-month follow-up (Table 6). After the 3-month follow-up 22 participant's scores in regards to suspecting CSA increased, 141 stayed the same, and 23 decreased.

**Table 6:** **Behavior change results**.

	**Median**		**Range**		***Z*-score**	***p*-value**	**Effect Size^a^**
	**Baseline**	**3 Month Follow-up**	**Baseline**	**3 Month Follow-up**			
Number of times talking about CSA and HSD	0	1	3	3	2.456	0.014^*^	0.18
Total number of topics discussed	2	4	11	11	3.204	0.001^**^	0.23
Number of times suspecting CSA	0	0	3	3	0.250	0.802	NS^b^
Number of times reporting CSA	0	0	2	2	0.179	0.858	NS
Total number of individual action strategies used	1	2	3	3	5.398	<0.0005^***^	0.39
Being a responsible role model for other adults	1	1	1	1	2.926	0.003^*^	0.21
Watching for signs of abuse in children	0	1	1	1	5.830	<0.001^***^	0.42
Taking steps to protect children	0	0	1	1	3.736	<0.001^***^	0.27
total number of organizational action strategies used	2	6	9	9	4.165	<0.0005^***^	0.37

*CSA, child sexual abuse*.

*HSD, healthy sexual development*.

a *Effect sizes based on Cohen's (1992), r = 0.01 (small effect), r = 0.30 (medium effect), r = 0.50 (large effect)*.

b *NS = non-significant. Effect sizes were not calculated when results were non-significant*.

* *Results significant at p = 0.05*.

** *Results significant at p = 0.001*.

*** *Results significant at p < 0.001*.

#### Reporting child sexual abuse

A total of 180 study participants completed both baseline and 3-month follow-up questionnaires on this section of the behavioral questionnaire. Results of a Wilcoxon signed-rank test found no statistically significant changes between the median at baseline and 3-month follow-up (Table 6). After the 3-month follow-up, 13 participant's scores in regards to reporting CSA increased, 155 stayed the same, and 12 decreased.

#### Number of times talking about child sexual abuse or healthy sexual development

A total of 186 study participants completed both baseline and 3-month follow-up questionnaires in this section of the behavioral questionnaire. Results of a Wilcoxon signed-rank test found a statistically significant change between the median at baseline and 3-month follow-up (Table 6). From the total number of 186 participants 81 scores increased, 51 scored did not change, and 54 scores decreased.

#### Total number of topics talked about

**Figure 2:**
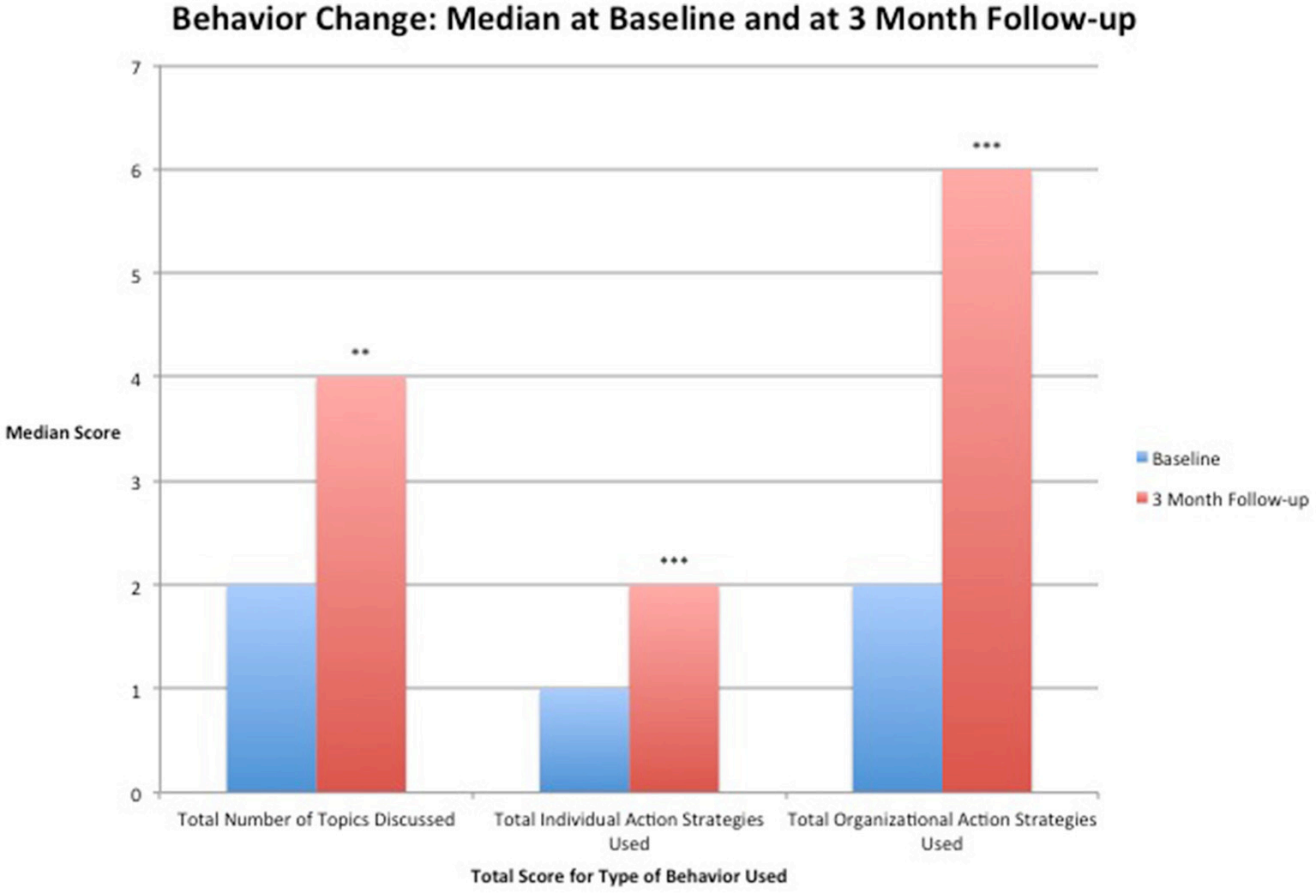
**Median score for specific behavior change categories before taking the workshop (baseline) and at 3-months after taking the workshop (3-month follow-up)**. ^**^Results significant at *p* = 0.001. ^***^Results significant at *p* < 0.001.

A total of 195 study participants completed both baseline and 3-month follow-up questionnaires on this section of the behavioral questionnaire. The median score at 3-month follow-up was higher than at pre-test. Results of a Wilcoxon signed-rank test found a highly statistically significant change between the median at baseline and 3-month follow-up (Table 6, Figure 2). From the total number of 195 participants there were 101 increased scores, 31 did not change, and 63 had decreased scores.

#### Total individual prevention strategies used

A total of 194 study participants completed both baseline and 3-month follow-up questionnaires on this section of the behavioral questionnaire. Median score at 3-month follow-up was double that at pre-test (Figure 2). Results of a Wilcoxon signed-rank test found a statistically significant change between the median at baseline and 3-month follow-up (Table 6). Of the 194 participants that completed these measures, 101 had an increase in score, 49 scores did not change, and 44 scores decreased.

**Figure 3:**
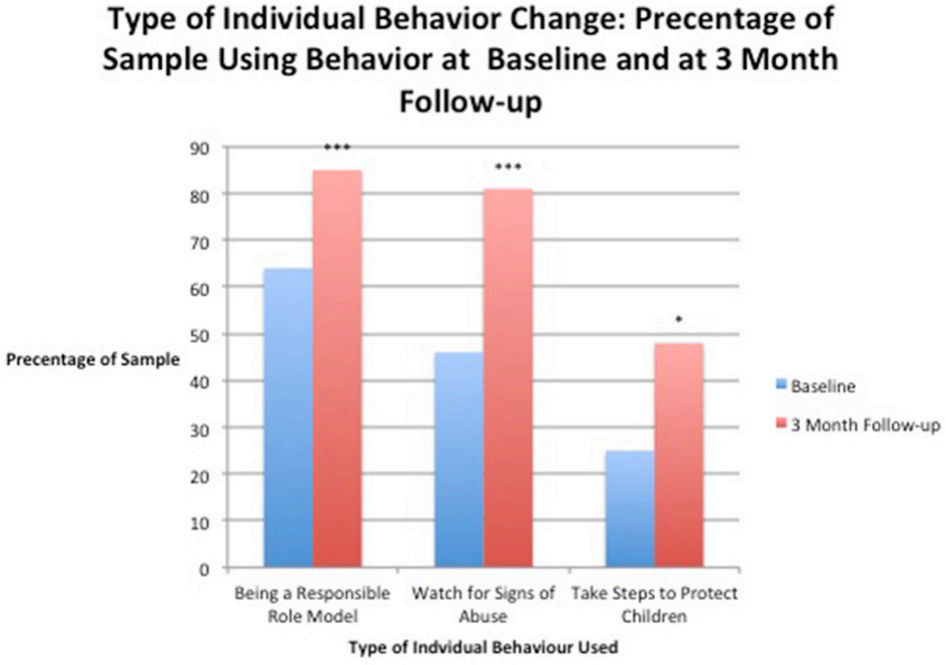
**Percentage of the sample that reported using each individual prevention strategy before taking the program (baseline) and after taking the program (3-month follow-up)**. ^*^Results significant at *p* < 0.05. ^***^ Results significant at *p* < 0.001.

The individual action strategies are shown in Figure 3 with each category being measured using the percentage of the sample that used each strategy both at baseline and at 3-month follow-up (Table 7). Results of a Wilcoxon signed-rank test found a statistically significant change for watching for signs of abuse between the median at baseline and 3-month follow-up (Table 6). Of the 194 participants that completed these measures, 72 had an increase in score, 105 scores did not change, and 17 scores decreased. The Wilcoxon signed-rank test also found statistically significant change for taking steps to protect children from baseline to 3-month follow-up. Of the 194 participants that completed these measures, 74 had an increase in score, 85 scores did not change, and 35 scores decreased. Statically significant change from pre-test to 3-months follow-up also occurred in being a responsible role model for other adult. Of the 194 participants that completed these measures, 74 had an increase in score, 85 scores did not change, and 35 scores decreased.

**Table 7:** **Behavioral measures expressed as percentages**.

**Behavioral Measures**	**Percentage of Sample**	
	**Baseline**	**3 Month Follow-up**
NUMBER OF TIMES TALKING ABOUT CSA And HSD^a^^*^		
0 times	51.0	28.2
1–2 times	27.4	38.8
3–4 times	11.6	19.7
5 or more times	10.0	13.3
TYPES OF TOPIC DISCUSSED^b^^**^		
Boundaries	66.0	76.4
Identifying a range of emotions	44.6	56.4
Internet safety	31.4	41.5
Proper names for genitals	36.3	46.7
Using the word “surprise” for things like birthday presents rather than “secret”	15.1	40.5
Definition of sexual abuse	10.3	12.3
Grooming techniques adults might use	8.3	14.4
Children are never to blame if they are sexually abused	13.8	22.6
What to do if you are sexually abused	14.7	20.5
How to tell someone if you are sexually abused	14.1	17.9
Saying “no” is allowed	43.3	63.1
**NUMBER OF TIMES SUSPECTING CSA^a^^, NS^**		
0 times	84.8	86.1
1-2 times	11.6	11.8
3-4 times	1.6	1.1
5 or more times	1.9	1.1
**Number OF TIMES REPORTING CSA^a^^, NS^**		
0 times	92.7	92.5
1-2 times	6.9	7.0
3-4 times	0.3	0.5
5 or more times	0	0
INDIVIDUAL ACTION STRATEGIES USED^a^^***^		
Being a responsible role model for other adults	26.6	7.2
Watching for signs of abuse in children	30.4	14.4
Taking steps to protect children	24.7	35.6
ORGANIZATIONAL ACTION STRATEGIES USED^b^^***^		
Criminal record checks	55.9	88.5
Child welfare checks	40.7	63.9
Screening interviews	30.8	58.2
Reference checks	41.7	72.1
Provide written policy outlining appropriate conduct with children	23.1	55.4
Monitoring one-on-one time between adults and children	20.8	48.4
Provide written policy for handling suspicions of abuse	24.4	47.5
Provide written policy for handling disclosure of abuse	24.0	47.5
Provide written policy for identifying and handling inappropriate comments and behaviors by adults	18.6	45.1

*This table shows descriptive data for each behavioral measure separated into individual variables using the percentage of the total sample at baseline and at 3 month follow-up*.

a *Participants were asked to select all that applied therefore percentages in these categories total more than 100*.

b *Participants were able to select only one answer therefore percentages in these categories equal 100*.

* *Results significant at p = 0.05*.

** *Results significant at p = 0.001*.

*** *Results significant at p < 0.001*.

#### Total organizational prevention strategies used

A total of 124 study participants completed both baseline and 3-month follow-up questionnaires on this section of the behavioral questionnaire, with the rest stating this section of the questionnaire was not relevant to their role. Median scores at 3-month follow-up were three times higher than at pre-test (Figure 2). Results of a Wilcoxon signed-rank test found a highly statistically significant change between the median at baseline and 3-month follow-up (Table 6). Of these 124 individuals involved with organizations 79 had an increase in score, 9 scores did not change, and 37 scores decreased.

### Descriptive statistics and behavioral change measures

Behavioral measures were also separated into individual variable categories and looked at using percentages of the total sample (Table 7) in order to gain more insight into changes that occurred in the 3 months after taking the program. We ran statistical analysis on the variables included within individual action strategies (Table 6) because these variables were of particular interest. We did not run analysis to determine the statistical significance the remainder of these categories because of the high number of variables and associated increasing risk of Type I errors as more tests are run.

### Correlations between outcome variables and demographic variables

Using a Spearman's rank correlation co-efficient test we examined potential correlations between demographic variables and changes in knowledge attitude, total topics talked about, total individual action strategies used, total organizational strategies used, number of times suspecting CSA, or number of times reporting CSA. However, we found no statistically significant correlations between these variables and gender, age, highest level of education completed, or self-reported sexual abuse as a child other than a single negative relationship between having previous CSA training and change in use or organizational action strategies. There was a significant negative relationship between having previous CSA training and change in use or organizational action strategies, *r*_s_ = −0.278, *p* = 0.002.

### Effect sizes

Medium effect sizes were seen for individual and organizational behavior change and small effect sizes for measures related to talking with children about CSA and HSD (Table 6). Effect size for knowledge change was large (0.57) and medium for attitude (0.34) (Table 5).

## Discussion

The *Prevent It!* program workshops described in this study are a novel approach to create a program that can potentially help, or even reduce, the large numbers of individuals who experience CSA. This program was developed in a comprehensive manner and is unique compared to previous approaches. The goal was to develop an evidence-based program that can be administered by community volunteers adults involved with children, which will increase their knowledge and attitudes toward CSA. Perhaps even more importantly, the primarily outcome goal was that the *Prevent It!* program would improve the actual behaviors of the adults taking part. The results from the present study strongly support these intentions. The results clearly show that this program significantly improves knowledge, attitudes, and behaviors in adults that take the program.

Interestingly, while we found many changes overall, for the two items, “Suspecting CSA” and “Reporting CSA,” there were no statistically significant changes. However, we believe the likely reason for this lack of change was that 3-months was not enough time for individuals to be in situations where they may have come across CSA. To answer such a question, a 12-month follow-up may help determine if the *Prevent It!* program changes these behaviors over the longer-term, and we anticipate such longer-term research in the future.

Our findings contribute to a small and growing field of research regarding adult-targeted CSA prevention. Results support previous studies that have found classroom-based teaching of adults about CSA can improve knowledge, attitudes, and behavioral intentions. However, one drawback of classroom-based training is the cost involved, and therefore internet based approaches are now starting to be considered because they are more cost-effective and can increase accessibility (Wurtele, 2009). It is uncertain about differential effectiveness of in-person training with a facilitator (Paranal et al., 2012; Rheingold et al., 2012) although results from one novel study suggest that an online version can be as effective as a classroom version for childcare professionals (Rheingold et al., 2015). Ideal future research will involve comparisons with an internet-based version of *Prevent It!* to assess if this is true for general public adults as well. For these reasons, we are developing this, and plan to study its effectiveness in future research. Additionally, although the program is designed to be facilitated by community volunteers, the present study utilized experienced staff, and therefore further research should compare the outcomes when such volunteers are facilitators.

Nonetheless, despite the positive findings from the present study, there are some methodological matters that should be considered as potential limitations. Firstly, we utilized a within-subject design comparing baseline to 3-month follow-up, with studies suggesting that attitudes and behavioral intentions are persistent over time (Deblinger et al., 2010). Nonetheless, for further certainty that all these positive findings were due to the program and not other factors, a randomized wait-list control study could be carried out. Secondly, our study population only consisted of 63% of those who took the program and completed baseline demographic data. However, there were no statistically significant differences in demographics between those who completed follow-up and those who did not, and we therefore believe that the results reflect the wider group, not just the responders. Thirdly, only a small number of men (*n* = 22) took part in the program, and therefore the impact of this program on men is less certain. This is a problem that is common in studies in this area (i.e., Rheingold et al., 2012, 2015), and it has also been suggested that there may be less support regarding the issue of CSA in some male-dominated organizations (Parent and Demers, 2009). It is possible that subsequent focus group research involving men may help address this issue. Fourthly, next steps in this field of research are to address challenges associated with the limitations of the measurement tools used. Cronbach's alpha may be a useful measure of internal consistency moving forward and some assessment of reliability and validity of the measurement tools will the generalizability of results in this study and others. Also, because self-report measures of behaviors were used, results may be biased by social desirability. The anonymous online questionnaire format of the measures may help to reduce the impact of social desirability in this study.

In conclusion, the purpose of this study was to determine the effectiveness of a CSA prevention program. Previous research has demonstrated the ability of programs such as this to change knowledge and increase participant's likelihood of using preventative behaviors. This is the first study to measure general public adult's self-reported use of preventative behaviors. Compared to baseline, at the 3-month follow up, participants who took the program were talking about CSA and healthy sexual development twice as much, were using twice as many individual action strategies, and were utilizing three times as many organizational strategies. Knowledge and positive attitude also increased significantly. The results suggest that the *Prevent It!* program is highly effective, and strongly support it's more widespread use.

## Author contributions

PS and EM designed and performed all aspects of the study.

### Conflict of interest statement

Previously, PS has been a non-paid volunteer on the Governance Board of Little Warriors. Neither author has any financial interest in the *Prevent It!* program. The other author declares that the research was conducted in the absence of any commercial or financial relationships that could be construed as a potential conflict of interest.
